# Varroa‐Sensitive Hygiene (VSH) as a Potential Contributor to the Natural Survival of Ethiopian Honey Bees (*Apis mellifera simensis*) Against *Varroa destructor* Mite

**DOI:** 10.1002/ece3.74461

**Published:** 2026-10-16

**Authors:** Alemu Tsegaye, Tessema Aynalem, Mengistie Taye, Workneh Ayalew, Abebe Jenberie, Mary Chege, Tadelle Tefera, Beatrice Nganso

**Affiliations:** ^1^ Agricultural Biotechnology Division, Institute of Biotechnology Bahir Dar University Bahir Dar Ethiopia; ^2^ Sekota Dryland Agriculture Research Center Sekota Ethiopia; ^3^ International Centre of Insect Physiology and Ecology (ICIPE) Nairobi Kenya; ^4^ Organization for Rehabilitation and Development in Amhara (ORDA) Bahir Dar Ethiopia

**Keywords:** *Apis mellifera simensis*, Ethiopia, *Varroa*‐sensitive hygiene

## Abstract

Although the *Varroa destructor* mite is the primary threat to honey bee (*Apis mellifera*), populations of *A. m. simensis* in Ethiopia naturally survive mite infestations without treatment. While general hygienic and grooming behaviors have been previously reported to contribute to the natural survival of this subspecies against Varroa mites, the role of Varroa‐sensitive hygiene (VSH) remains unknown. Here, we experimentally evaluated four VSH responses in worker brood cells of this subspecies artificially infested with a single mite (treatment) or opened and resealed without introducing a mite (control) across varying infested patch sizes (five, eight, and ten neighboring cells): untouched cells (UC), cell cap opened or partially opened (CO), brood and introduced mite removed (BMR), and introduced mite removed, brood retained, and cell recapped (MR). Our results revealed that VSH responses differed significantly between treatment categories and were significantly dependent on infested patch size. At five‐cell patch size, VSH responses did not differ between treatment categories, whereas eight‐ and ten‐cell patch sizes significantly increased the odds of BMR, MR and/or CO relative to UC by between five‐ and twenty‐nine‐fold. Model‐predicted probabilities showed that the removal response (BMR + MR) accounted for 64.9% and 83.7% in infested patches at eight and ten cells, respectively, compared with 21.5% and 23.1% in controls. The uncapping response (CO + BMR + MR) reached 71.2% and 89.8% in eight and ten cells compared with 33.1% and 30.2% in controls, respectively. Overall, these findings suggest VSH as an additional behavioral mechanism of Ethiopian honey bees against *V. destructor*.

## Graphical abstract

This study investigated *Varroa*‐sensitive hygiene (VSH) in worker brood cells of *Apis mellifera simensis* artificially infested with a single *Varroa destructor* mite. Removal responses reached 64.9% and 83.7% in patches of eight and ten neighbouring infested cells, respectively, compared with 21.5% and 23.1% in controls, while uncapping responses reached 71.2% and 89.8%, respectively, compared with 33.1% and 30.2% in controls. These findings suggest that VSH is an additional behavioural mechanism contributing to the natural survival of Ethiopian honey bees against *Varroa* mites.

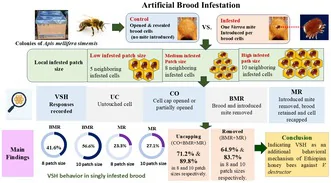

## 1 Introduction

The invasive ectoparasitic mite, *Varroa destructor* , is the most serious threat to honey bee ( *Apis mellifera* ) health in numerous countries (Martin et al. 2012; Traynor et al. 2020; Morfin et al. 2023). The mite feeds on the fat body and haemolymph of adult and immature honey bees (Ramsey et al. 2019; Han et al. 2024), leading to weight loss, reduced flight performance, immune suppression, and shortened lifespan (Amdam et al. 2004; Kralj and Fuchs 2006; Yang and Cox‐Foster 2005; Aronstein et al. 2012). In addition, *Varroa* mite vectors deadly viruses to honey bees, altering viral dynamics within colonies and increasing colony‐level impacts (Mondet et al. 2014; Boecking and Genersch 2008). Current management approaches rely on a combination of chemical, physical and selective breeding strategies, as no single approach has provided a sustainable solution to the mite problem to date; however, their effectiveness, benefits, and associated trade‐offs vary (Locke 2012; Guichard et al. 2020; Lester 2023; O'Connell et al. 2025).

Several studies have shown that, unlike mite‐susceptible European honey bee populations, the genetically diverse African honey bee subspecies are resilient to *Varroa* mite infestations without requiring any interventions at the colony level. This resilience has been attributed to a suite of adaptive behavioral traits, including general hygienic behavior (GHB) (Gebremedhn et al. 2019; Wanore, Pirk, Yusuf, Ayalew, and Nganso 2025), grooming behavior (Boecking and Spivak 1999; Nganso et al. 2017), suppression of mite reproduction in worker brood cells (Nganso et al. 2018; Gebremedhn et al. 2019), and *Varroa*‐sensitive hygiene (VSH) (Cheruiyot et al. 2018). In addition, African honey bees appear capable of tolerating deadly viruses vectored by the mites, potentially due to enhanced hygienic behavior (De Souza et al. 2021). Moreover, proteins upregulated in mite‐tolerant African honey bees are reported to be linked to immunity or stress responses to mite infestations (Fang et al. 2022).

Ethiopian honey bees, *A. m. simensis* (Meixner et al. 2011; Hailu et al. 2020; Wanore, Pirk, Yusuf, Baleba, et al. 2025), have also been reported to exhibit several behavioral traits associated with reduced *Varroa* impact, including low mite reproductive success in worker brood cells (0.27) compared to values reported for European (1.01) and Africanized bees (0.64) (Gebremedhn et al. 2019), high GHB (Gebremedhn et al. 2019; Wanore, Pirk, Yusuf, Ayalew, and Nganso 2025), and grooming behavior (Wanore, Pirk, Yusuf, Baleba, et al. 2025). However, their ability to express *Varroa*‐sensitive hygiene (VSH) remains unexplored. This behavior is a specialized form of GHB directed specifically at mite‐infested brood (Harris 2007) and is triggered by olfactory cues emitted by parasitized worker brood (Schöning et al. 2012; Mondet et al. 2015). It is a heritable behavioral trait that involves a sequence of responses to interrupt the mite's reproductive cycle within infested brood, including detection, uncapping, brood removal, mite removal and brood‐cell recapping (Boecking et al. 2000; Harris 2007). VSH has been previously documented in several mite‐surviving honey bee populations, including those found in Kenya (Cheruiyot et al. 2018), Russia (Kirrane et al. 2015), the Arista/Netherlands (Panziera et al. 2017), and the USA (Harris 2007).

In this study, we therefore investigated VSH behavioral responses of *A. m. simensis* colonies to artificially infested worker brood cells with a single mite. Colonies were first screened for GHB, and those exhibiting high GHB (> 90% pin‐killed brood removal within 24 h) were subsequently selected for the VSH assessment. We compared VSH behavioral responses between singly mite‐infested (treatment) and sham‐manipulated (control) brood cells and examined the effect of local infestation patch size on the expression of these responses. By experimentally characterizing VSH‐related responses in *A. m. simensis*, this study provides new information on the expression of this behavioral trait and its potential contribution to the natural survival of Ethiopian honey bees against *Varroa* mite infestations.

## 2 Materials and Methods

### 2.1 Study Site

The research study was conducted at the Jinkaba experimental apiary of the Sekota Dryland Agricultural Research Center (SDARC) in the Amhara region, Ethiopia. The apiary is located at 12°41′01.02″ N latitude and 39°00′31.53″ E longitude. The study was conducted between August and November 2024, which corresponds to the active season of colony development.

### 2.2 General Hygienic Behavior of *A. m. simensis* Colonies

From the apiary, 20 colonies of *A. m. simensis*, headed by naturally mated queens, were randomly selected and screened to identify 10 colonies exhibiting high levels of general hygienic behavior (GHB). Colonies were maintained in standard Zander hives containing eight to ten brood combs and were placed on stands protected by oil‐filled cans to prevent ant infestation. The GHB of each colony was assessed using the pin‐kill brood assay as described before (Wanore, Pirk, Yusuf, Ayalew, and Nganso 2025). Briefly, a section of a brood frame containing 100 capped worker brood cells at the purple‐eyed stage was marked, and each cell was pierced using a sterile #2 entomological pin. The colony's GHB, quantified as the percentage of fully removed pin‐killed cells, was assessed 24 and 48 h later based on the examination of photographs of the assay area taken at 0, 24, and 48 h. The GHB was calculated using the following formula (Cheruiyot et al. 2018):

$$\%\mathrm{GHB}=\left(\frac{N_{\mathrm{rem}}}{N_{\mathrm{tot}}-N_{\text{uncap}}}\right)\times 100$$

where: $\begin{array}{c} N_{\mathrm{rem}}=\text{Total number of fully}\;\text{removed cells in the assay area at}\;24 \end{array}$ or48h; $N_{\mathrm{tot}\;}=\text{Total number of pinned}\;\text{cells in the assay area}\;\left(100\;\text{cells}\right)$; $\begin{array}{c} N_{\text{uncap}}=\text{Total number ofcells already uncapped or emptied in the} \end{array}$ assay area at time0h.

Thus, cells that were already uncapped or cleaned/emptied at 0 h in the assay area were excluded from the calculation. No partial removal of brood was observed in the assay area at 24 or 48 h; therefore, only fully removed cells were counted as removed. Among the colonies that removed more than 90% of the pin‐killed worker brood and were considered highly hygienic at 24 h, ten colonies were randomly selected for subsequent assessment of VSH behavior (Cheruiyot et al. 2018; Pereira et al. 2013).

### 2.3 *Varroa*‐Sensitive Hygiene (VSH) in Colonies of *A. m. simensis*

Detailed methods for evaluating VSH behavior have been described in previous studies (Aumeier et al. 2000; Panziera et al. 2017; Cheruiyot et al. 2018). Briefly, from each of the ten colonies selected, three frames with approximately 200–300 uncapped worker brood larvae at the 5th larval instar were randomly selected. The fifth‐instar larvae were visually identified by their stretched, elongated body posture, pearly‐white cuticle, and prominent segmental rings, characteristic of the immediate pre‐capping stage, rather than the tightly coiled “C” shape observed in earlier instars (Farris et al. 1999; Anderson et al. 2023). The selected frames were marked using a permanent marker pen. A transparent plastic sheet was overlaid on each frame, and the positions of the 5th larval instar were marked on the sheets using a permanent marker pen. The frames were subsequently returned to their original colonies, and the marked brood were monitored until cell capping. Within 6 h after capping, the marked frames were collected from the colonies, and the previously marked brood were located using the transparent plastic sheets. The edges of the selected capped worker brood cells were carefully opened using sterile surgical blades. Female phoretic or non‐reproducing *Varroa* mites were collected from five naturally infested colonies using the standard sugar shake method 2 h before inoculation (Dietemann et al. 2013), and only active mites, defined by rapid movement upon tactile stimulation with a fine paintbrush, were chosen in all the experiments as described before (Nganso et al. 2020). These colonies were unrelated to those used for GHB and VSH assessment.

To evaluate the effect of local infestation patch size on the expression of VSH behavior, three infested patch‐size treatments were evaluated by artificially infesting patches consisting of five, eight, or ten neighboring cells. Patch size was defined by the number of singly infested cells: 5, 8, or 10 neighboring cells. Because honey bee brood cells are arranged in a hexagonal pattern, neighboring cells were selected to form compact, approximately circular patches to standardize patch geometry for each patch‐size treatment group while accommodating the natural arrangement of cells within the comb. Each selected brood within a patch was infested with a single mite using a fine paintbrush. At each timepoint, a single frame per colony (A, B, or C) received one of the three infested patch‐size treatments. On the opposite side of the same frame, an equal number of cells were sham‐manipulated (opened and resealed without mite introduction), maintaining a 1:1 ratio of infested to control cells (e.g., five infested and five sham‐manipulated cells). This spatial separation minimized potential cross‐contamination or behavioral interference, such as mite transfer or localized hygienic responses, between infested and control cells. Treatment assignment was rotated across six sequential time points (TP1‐TP6), each separated by 72 h, such that each colony received each of the three patch‐size treatments (five, eight and ten neighboring infested cells) twice during the study period. The complete randomization and rotation schedule is provided in Table S1. The exact locations of infested and sham‐manipulated cells were recorded on the transparent plastic sheets overlaid on each comb. At each time point, new, previously unmanipulated fifth‐instar larvae were selected on the designated frame using the morphological criteria described above. The selected brood cells were monitored until natural capping, and the marked cells were subsequently located within 6 h of capping. Freshly capped worker brood were identified by the presence of dull, light‐to‐medium brown or tan cappings, compared with the darker, more weathered or discolored capping of older brood. The capped cells were then carefully opened for mite introduction. Thus, although the same physical frames were reused across time points, different, previously unmanipulated brood cells were selected at each time point, and only freshly capped brood was used for mite introduction.

After inoculation, frames were returned to their original colonies to allow worker honey bees to seal cells properly. After 24 h, these frames were inspected to assess the success of the manual infestation (acceptance rate) by recording the number of manipulated cells that remained intact and were not removed by worker bees. Cells that remained intact after 24 h were considered successfully infested and were subsequently evaluated at 72 h, following Panziera et al. (2017). At 72 h postinoculation, the marked brood cells were evaluated to record the following behavioral responses: untouched cells (UC) with original silk cap remaining intact, cell cap opened or partially opened (CO), brood and introduced mite removed (BMR), and introduced mite removed, brood retained and cell recapped with fresh wax (MR) (Oddie et al. 2018; Martin et al. 2020). Based on their relationship to classical *Varroa*‐sensitive hygienic behavior, the uncapping response was defined as CO + BMR + MR, while the removal response was defined as BMR + MR (Cheruiyot et al. 2018). In sham controls, these categories described brood and cell responses only and were not interpreted as mite removal because no mite was introduced. Specifically, BMR indicated brood removal, MR indicated brood retention with recapping, UC indicated an untouched sealed cell, and CO indicated a cell with the cap opened or partially opened. In total, 460 active mites were introduced into brood cells, and 460 additional cells were sham manipulated as controls without introducing any mites. Endpoint scoring was not blinded, as the observer conducted on‐site field tracking and data recording using transparent sheets overlaid on the brood comb to identify individual brood cells and the observer was aware of their treatment and control status.

For VSH assessment, each target cell was carefully uncapped under bright illumination from a head torch and using a magnifying glass, and the pupa was gently removed with fine forceps. The removed pupa was thoroughly examined by rotating it to inspect its body surface for *Varroa* mites and mite offspring. The interior walls and bottom of the cell were also carefully inspected for mites and associated signs of mite presence. Cells were therefore classified as having the introduced mite removed when the introduced mite was not observed on the pupa, cell walls, or cell bottom during the inspection. To assess recapping, the wax cap of each target brood cell was carefully opened using fine forceps, with the cap preserved for examination. The underlying cocoon was inspected to distinguish undisturbed from recapped cells. Undisturbed cells retained an intact, glossy spun‐silk cocoon layer, whereas recapped cells showed a disrupted or absent glossy cocoon layer and a newly repaired, duller wax cap composed of wax particles. The recapping status of each cell was recorded together with its brood and mite outcome.

### 2.4 Statistical Analysis

All statistical data analyses were performed using R‐software version 4.5.1 (R Core Team 2025). General hygienic behavior was analyzed separately at 24 and 48 h using binomial generalized linear models (GLMs) with a logit link function. Colony was included as a categorical explanatory variable to assess differences in GHB among colonies at each time point. Model significance was assessed using analysis of deviance with a chi‐square test. Inter‐colony variability in GHB at each time point was assessed descriptively by calculating the variance of the percentage‐removal data using the *var()* function in R‐software (R Core Team 2025). Box plots with superimposed jitter were used to illustrate the distributions and highlight variation at the colony level using the dplyr (Wickham et al. 2026) and ggplot2 (Wickham 2016) packages.

To account for the hierarchical and paired experimental design, a multinomial mixed‐effects model was fitted using the *mblogit()* function in the *mclogit* package (Elff 2022), with marginal quasi‐likelihood (MQL) as the estimation method. The model included treatment group (control versus infested), patch size treatment (five, eight, or ten neighboring infested cells), and time of manipulation (TP1–TP6) as fixed effects, alongside the interaction term between treatment group and patch size treatment. A random intercept was included for the frame‐pair unit, defined as the combination of colony, time of manipulation, and frame ID (colony × time of manipulation × frame ID), to account for the pairing of control and infested patches on the same frame and repeated observations within colonies. The four mutually exclusive behavioral outcomes (UC, CO, BMR, and MR) were retained as multinomial response categories, with UC as the reference category. The potential interaction between treatment group and time of manipulation was also evaluated by fitting an expanded model with the same multinomial response structure and random‐effects specification. However, the expanded model failed to converge, and the interaction was therefore not retained in the final model. Because the treatment group × time interaction could not be reliably evaluated owing to non‐convergence of the expanded model, the final model retained time of manipulation as a main effect. Model‐predicted probabilities for treatment group and patch size were therefore summarized across the six timepoints (TP1–TP6), while the timepoint main effect was assessed and reported separately. The significance of fixed effects was assessed using analysis‐of‐deviance tests comparing the full model with reduced models that excluded each predictor of interest. Statistical significance was determined from the difference in model deviance and the corresponding degrees of freedom. Because the interaction between treatment group and patch size was significant, comparisons of the behavioral responses (CO, BMR, and MR relative to UC) between control and infested cells were carried out separately at each patch size treatment. Odds ratios (ORs) and 95% confidence intervals were calculated from the corresponding log‐odds contrasts, and *p*‐values were Bonferroni‐adjusted across the nine planned comparisons. Model‐predicted probabilities were also calculated for the four VSH responses and were used to derive the removal response (BMR + MR) and the uncapping response (CO + BMR + MR).

## 3 Results

### 3.1 General Hygienic Behavior (GHB) of *A. m. simensis* Colonies

Of the 20 colonies examined for GHB at 24 h, 12 colonies (60%) exhibited high hygienic behavior (greater than 90% removal rate) (Figure 1). At 24 h, GHB differed significantly among colonies (Binomial GLM: *χ* ^2^ = 90.37, df = 19, *p* < 0.001). By 48 h, all colonies showed greater than 90% brood cell removal rate (Figure 1), with mean GHB increasing from 87.58% ± 1.63% at 24 h to 98.03% ± 0.37% at 48 h. Although minor inter‐colony variation persisted as colonies approached full removal at 48 h (Binomial GLM: *χ* ^2^ = 31.59, df = 19, *p* < 0.05), overall GHB variation was substantially higher during the 24‐h screening window (variance = 53.31; range 70.97%–100%) than at 48 h (variance = 2.70; range: 95.30%–100%).

**FIGURE 1:**
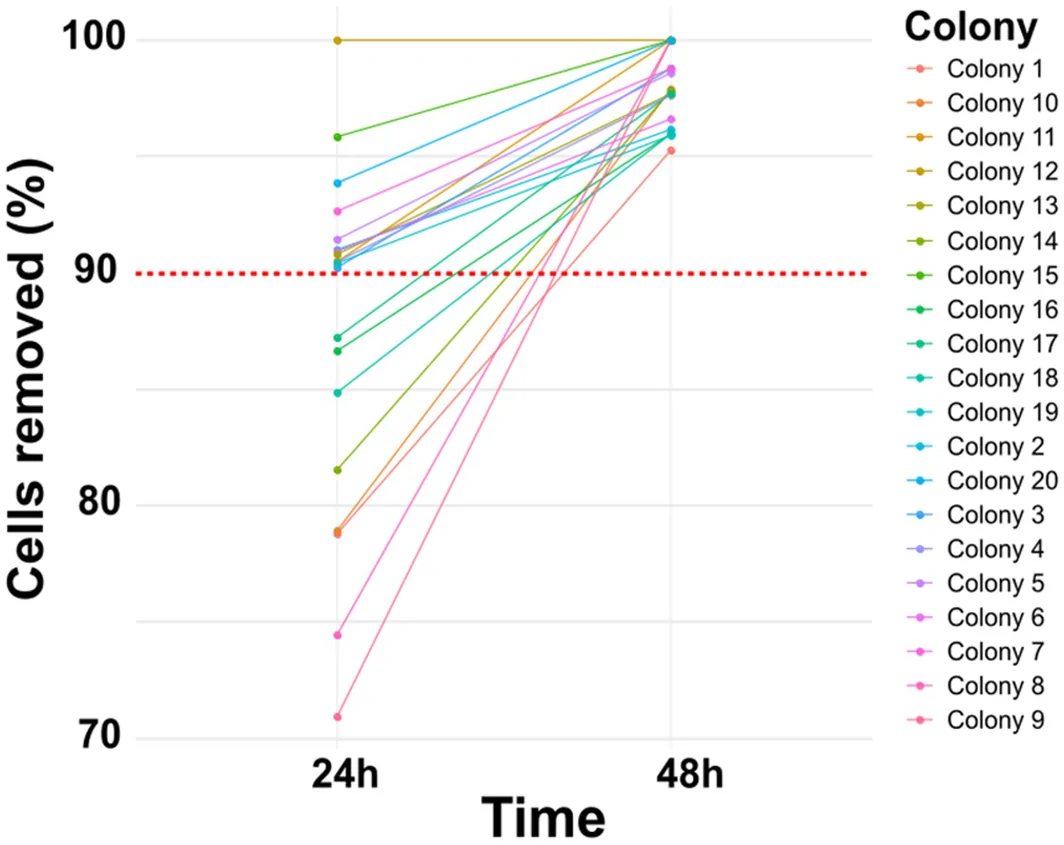
Spaghetti plot showing variability in the general hygienic behavior (GHB) among honey bee colonies at 24 h and 48 h.

### 3.2 *Varroa*‐Sensitive Hygiene (VSH) in Colonies of *A. m. simensis*

Of the 920 total artificial inoculation attempts across ten *A. m. simensis* colonies, 816 cells (411 control and 405 artificially infested) met retention criteria at 24 h postinoculation and were therefore evaluated at 72 h endpoint. After 72 h, we observed the four categories of behavioral responses investigated in colonies of *A. m. simensis*: untouched cells (UC), cell caps opened (CO), brood and introduced mite removed (BMR), and introduced mite removed, brood retained and cell recapped (MR).

The multinomial mixed‐effects model showed that the behavioral response profile differed between control and infested cells (deviance difference = 207.78, df = 9, *p* < 0.001). The effects of patch size treatment (deviance difference = 144.74, df = 12, *p* < 0.001) and time of manipulation (deviance difference = 106.11, df = 15, *p* < 0.001) were also significant. Moreover, the interaction between treatment groups (control versus infested cells) and patch size was significant (deviance difference = 65.39, df = 6, *p* < 0.001). The full model provided a significantly better fit than the null model (deviance difference = 376.47, df = 30, *p* < 0.001).

Pairwise analyses showed that worker response to infestation became significantly stronger with increasing patch size. At the five‐cell patch size, none of the individual responses (CO, BMR or MR) differed significantly between control and artificially mite‐infested brood cells following Bonferroni correction, using UC as the reference category (*p* > 0.05). At the eight‐cell patch size, infestation significantly increased the odds of BMR relative to UC by approximately fivefold (OR = 5.40, 95% CI = 2.92–9.99, Bonferroni‐adjusted *p* < 0.001) and the odds of MR by approximately 27‐fold (OR = 26.68, 95% CI = 7.73–92.12, Bonferroni‐adjusted *p* < 0.001), whereas CO rates did not differ significantly (*p* > 0.05). At the ten‐cell patch size, infestation significantly increased the odds of all three behavioral outcomes relative to UC: CO (ORs = 6.00, 95% CI = 2.33–15.47, Bonferroni‐adjusted *p* < 0.01), BMR (OR = 27.07, 95% CI = 14.18–51.66, Bonferroni‐adjusted *p* < 0.001), and MR (OR = 29.22, 95% CI = 13.05–65.43, Bonferroni‐adjusted *p* < 0.001).

Model‐predicted probabilities further confirmed this patch‐size‐dependent behavioral response (Table 1). At the five‐cell patch size, predicted probabilities of the four behavioral categories were broadly similar between control and infested patches (UC: 66.2% versus 56.8%; CO: 2.3% versus 5.9%; BMR: 29.2% versus 30.3%; MR: 2.3% versus 7.0%). At the eight‐cell patch size, the behavioral shift became more pronounced, with UC decreasing from 66.8% in control patches to 28.8% in infested patches, while BMR increased from 19.3% to 41.6% and MR from 2.2% to 23.3%, but CO remained relatively similar (11.6% vs. 6.3%). At the ten‐cell patch size, the response was strongest: UC decreased from 69.8% in control patches to 10.2% in infested patches, while BMR increased from 15.9% to 56.6%, and MR from 7.2% to 27.1%. The removal response (BMR + MR) was predicted at 31.5% in control and 37.3% in infested patches at the five‐cell patch size, increasing to 64.9% in infested patches compared with 21.5% in controls at eight cells, and to 83.6% compared with 23.1% at ten cells. Similarly, the uncapping response (CO + BMR + MR) was predicted at 33.8% and 43.2% in control and infested patches at five cells, respectively, increasing to 71.2% versus 33.1% at eight cells, and 89.8% versus 30.2% at ten cells.

**TABLE 1:** Model‐predicted probabilities (%) of VSH behavioral responses in control and artificially infested brood cells across varying patch sizes.

Treatment groups	Patch size	UC (%)	CO (%)	BMR (%)	MR (%)	Removal response (BMR + MR) (%)	Uncapping response (CO + BMR + MR) (%)
Control	5	66.2	2.3	29.2	2.3	31.5	33.8
Infested	5	56.8	5.9	30.3	7.0	37.3	43.2
Control	8	66.8	11.6	19.3	2.2	21.5	33.1
Infested	8	28.8	6.3	41.6	23.3	64.9	71.2
Control	10	69.8	7.1	15.9	7.2	23.1	30.2
Infested	10	10.2	6.1	56.6	27.1	83.7	89.8

Abbreviations: BMR, brood and introduced mite removed; MR, introduced mite removed, brood retained and cell recapped; BMR + MR, removal response; CO, cell cap opened or partially opened; CO + BMR + MR, uncapping response; UC, untouched cells with original silk cap remaining intact.

## 4 Discussion

### 4.1 General Hygienic Behavior (GHB) of *A. m. simensis* Colonies

Our study demonstrated that *A. m. simensis* from the Amhara region of Ethiopia expresses a strong general hygienic behavior (GHB), as all experimental colonies removed more than 90% of pin‐killed brood within 48 h. Notably, 60% of these colonies exceeded this threshold within 24 h, indicating considerable variation in the speed of hygienic responses among colonies. Consistent with previous findings on *A. m. simensis* from the same region, the 24‐h GHB assessment discriminated more effectively among colonies than the 48‐h assessment, at which point a ceiling effect in hygienic performance limited the detection of colony‐level differences (Wanore, Pirk, Yusuf, Baleba, et al. 2025). This substantial variation in hygienic performance at 24 h likely reflects differences in colony genetic composition, olfactory sensitivity to dead brood, and/or the proportion of worker bees within the appropriate age cohort available to perform hygienic tasks (reviewed in Spivak and Danka 2021). Rapid GHB is particularly relevant for resistance to brood pathogens and parasites because it minimizes opportunities for pathogen replication and parasite reproduction within colonies (Vung et al. 2020; Hristov et al. 2025; Amiri et al. 2026). Taken together, these findings suggest that the 24‐h assessment represents an effective benchmark for screening general hygienic traits among colonies of *A. m. simensis*.

The high levels of hygienic performance observed in *A. m. simensis* become even more evident when contrasted with pin‐killed brood removal/cleaning rates reported for other honey bee subspecies. In a 5‐year bidirectional selection study of *A. m. ligustica* populations in Israel, seasonal hygienic performance averaged approximately 52%–57% for cleaning (Seltzer et al. 2022), substantially below the more than 90% removal observed in most *A. m. simensis* colonies at 24 h in the present study. Similarly, Erez et al. (2022) reported an approximately 92% cleaning rate in *A. m. ligustica* colonies from Israel selected for high hygienic behavior, compared with 26% in low‐hygiene colonies. In contrast, unselected European colonies typically exhibit much lower cleaning rates. For instance, Balhareth et al. (2012) reported that unselected *A. m. carnica* colonies removed approximately 43.84% of pin‐killed brood, compared with an 85.28% removal rate by *A. m. jemenitica* in Saudi Arabia at 24 h. These comparisons highlight the exceptionally strong GHB expressed by Ethiopian honey bee populations, as shown in this and previous studies (Gebremedhn et al. 2019; Wanore, Pirk, Yusuf, Ayalew, and Nganso 2025), thereby strongly supporting the putative role of social immune defense in their natural survival against *V. destructor* infestations.

### 4.2 *Varroa*‐Sensitive Hygiene (VSH) in Colonies of *A. m. simensis*

In addition to the strong GHB observed, *A. m. simensis* exhibited pronounced *Varroa*‐sensitive hygiene, as workers responded significantly more to mite‐infested brood than to uninfested brood. The predominance of untouched cells (UC) in the control group compared to the artificially infested group indicates that worker responses were primarily associated with the presence of a live‐mite under our experimental conditions. However, this finding does not establish that the response was elicited uniquely by *Varroa*‐specific cues. As demonstrated by Sprau et al. (2023), dead or odor‐reduced mites trigger moderate removal responses, whereas pupae in cells containing live‐mites are removed significantly more frequently, indicating that the heightened response depends on additional live‐parasitism cues, such as odors from feeding wounds or distress‐related brood signals. Conversely, inorganic objects (e.g., glass beads) fail to elicit removal at rates above controls (Sprau et al. 2023), confirming that worker responses are not simply a generalized reaction to foreign‐object insertion. Consequently, the elevated responsiveness observed in our artificially infested cells is best interpreted as a live‐mite‐associated hygienic response, likely driven by a complex interplay between mite‐derived and brood‐associated cues. Previous studies have identified specific volatile compounds associated with *Varroa*‐parasitized brood that can trigger hygienic and recapping behaviors (e.g., octanoic acid, benzothiazole, n‐tetradecane, dodecanoic acid, and n‐hexadecane) (Noël et al. 2025), alongside antennal and central nervous system proteomic differences associated with VSH responsiveness (Mondet et al. 2015; Hu et al. 2016). Whether similar chemical dynamics, olfactory pathways, and/or neurobiological mechanisms underline the high VSH responsiveness observed in *A. m. simensis* warrants future investigation using comparative dead‐mite, live‐mites, brood‐wound or other cue‐specific controls.

The VSH behavioral profile of *A. m. simensis* workers showed a clear shift in behavioral responses following artificial mite infestation, particularly as the size of the infested brood patch increased. Significant effects of treatment group, infested patch size, and their interaction were found, with the strongest differences between control and infested groups occurring at eight‐ and ten‐cell patch sizes. Specifically, infestation increased the odds of brood and introduced mite removed (BMR) and introduced mite removed, brood retained and cell recapped (MR) relative to untouched cells (UC) by approximately 5‐fold and 27‐fold, respectively, at the eight‐cell patch size when compared with control patches. At the ten‐cell patch size, infestation increased the odds of cell cap opened or partially opened (CO), BMR, and MR relative to UC by approximately 6‐, 27‐, and 29‐fold, respectively. Model‐predicted probabilities further illustrate this response. At eight cells, the predicted probability of BMR increased from 19.3% in control patches to 41.6% in infested patches, while MR increased from 2.2% to 23.3%, respectively. At ten cells, BMR increased from 15.9% in controls to 56.6% in infested patches, while MR increased from 7.2% to 27.1%. Similarly, the removal response (BMR + MR) accounted for 64.9% and 83.6% of responses in infested patches at eight and ten cells, respectively, compared with 21.5% and 23.1% in control patches. Also, the predicted probability of the uncapping response (CO + BMR + MR) increased from 33.1% and 30.2% in controls to 71.2% and 89.8% in infested patches at eight and ten cells, respectively. The increase in response odds with increasing mite‐infested patch size demonstrates an innate behavioral capacity to scale social immune defenses as local infestation pressure intensifies. Similar mite‐infested patch‐size‐dependent effects have been reported in *A. m. scutellata* from Kenya (Cheruiyot et al. 2018) and VSH‐selected European populations from New Zealand (Kim et al. 2018), the USA (Wagoner et al. 2019) and Italy (De Iorio et al. 2025).

The pronounced VSH responses observed herein closely resemble those reported in naturally mite‐surviving *A. m*. *scutellata* from Kenya, where the overall removal response in singly infested worker brood cells averaged approximately 59% (BMR = 33% and MR = 26%) (Cheruiyot et al. 2018). They also closely resemble VSH responses reported in Africanized honey bees from Brazil (51%–61%, Guerra Jr et al. 2000), which are hybrids between *A. m. scutellata* from Africa and various European subspecies (Winston 1992), though lower removal rates have been documented in other Africanized populations (10%–25%, Aumeier et al. 2000). Taken together, the consistently high expression of VSH behavior observed across distinct African honey bee subspecies (*A. m. scutellata*, Africanized hybrids, and now *A. m. simensis*) suggests that they possess a robust hygienic response against *Varroa* mites. This capacity contrasts with mite‐susceptible European honey bee subspecies, where VSH behavior occurs at relatively low frequencies (25%–31%, Guerra Jr et al. 2000) and often requires selective breeding to reach substantial levels of expression (Harbo and Harris 2009; Danka et al. 2016). However, the levels of VSH behavior seen in African subspecies are comparable to those reported in certain isolated naturally mite‐surviving European strains, which are capable of removing up to 40% of singly mite‐infested brood cells (Kirrane et al. 2015; Mondet et al. 2015; Panziera et al. 2017). Notably, these removal responses remain well below the 99% removal observed in the mite's original host, the Asian honey bee *Apis cerana* (Rath and Drescher 2020), suggesting that while some *A. mellifera* populations exhibit strong VSH responses, they have not yet reached the level of resistance seen in the Asian honey bee.

The occurrence of introduced mite removal with brood retention and cell recapping (MR) in *A. m. simensis* colonies suggests that this behavior represents a complementary component of the broader VSH response against *Varroa* mite. Reported as a variant of VSH since it reflects the ability of workers to detect and respond to mites without sacrificing the developing brood (Boecking and Spivak 1999; Aumeier et al. 2000), it is worth noting that this behavior may not be exclusively associated with *Varroa* infestation. In fact, a recent study indicates that the presence, titer, and species of brood‐associated viruses may also influence recapping behavior in honey bees (Noël et al. 2026). Thus, the MR response observed in our study may reflect a response to a combination of *Varroa*‐associated and brood‐health‐related cues, rather than a response uniquely attributable to the introduced mite, warranting future investigation in colonies of this subspecies.

## 5 Conclusion

Overall, our study demonstrated that *A. m. simensis* workers express strong general hygienic behavior (GHB) and pronounced *Varroa*‐sensitive hygiene (VSH). Honey bee workers rapidly removed pin‐killed brood and responded selectively to mite‐infested brood mainly through brood and introduced mite removal (BMR) and introduced mite removal with brood retention and cell recapping (MR). Moreover, VSH responses increased with the local patch size of mite‐infested brood cells, indicating that *A. m. simensis* adjusts its social immune responses according to infestation pressure. Collectively, these findings suggest that VSH behavior could be an additional behavioral mechanism underlying the natural survival of Ethiopian honey bees against *Varroa destructor* . Future studies should elucidate the chemical, olfactory, and/or neurophysiological mechanisms underlying the high VSH responsiveness observed in *A. m. simensis* worker bees and distinguish the contributions of *Varroa*‐associated and brood‐health‐related cues using comparative dead‐mite, live‐mite, brood‐wound, viral‐status, and other cue‐specific controls. Although our behavioral categories were recorded only at the predefined 72‐h endpoint, cells removed before the 24‐h assessment may represent an early hygienic response. Future studies should also assess and record VSH responses at both 24 and 72 h to capture both early and late hygienic responses.

## Author Contributions

**Alemu Tsegaye:** data curation (lead), formal analysis (lead), investigation (lead), visualization (lead), writing – original draft (lead). **Tessema Aynalem:** conceptualization (equal), data curation (equal), formal analysis (equal), methodology (equal), writing – review and editing (equal). **Mengistie Taye:** conceptualization (equal), data curation (equal), formal analysis (equal), methodology (equal), writing – review and editing (equal). **Workneh Ayalew:** funding acquisition (lead), project administration (lead), writing – review and editing (supporting). **Abebe Jenberie:** writing – review and editing (equal). **Mary Chege:** writing – review and editing (equal). **Tadelle Tefera:** writing – review and editing (equal). **Beatrice Nganso:** conceptualization (lead), data curation (supporting), formal analysis (supporting), funding acquisition (equal), methodology (lead), supervision (lead), visualization (lead), writing – review and editing (equal).

## Funding

Data collection in Ethiopia and analysis at *icipe* was funded by the Mastercard Foundation through the More Young Entrepreneurs in Silk and Honey (MOYESH) Programme. Alemu T. Adane acknowledges the Bahir Dar University and Amahara Agricultural Research Institute (ARARI), FSRP Project for funding his PhD research work and studies.

## Ethics Statement

No ethical approval was required from the Ethics Committee of the International Centre of Insect Physiology and Ecology (*icipe*) because the study carried out in the field involved only invertebrate specimens (honey bees and *Varroa destructor* mites).

## Conflicts of Interest

The authors declare no conflicts of interest.

## Supporting information


**Data S1:** Code for analysis for this study.

**Data S2:** General hygienic behavior data.

**Data S3:** README.

**Data S4:** VSH data.

**Table S1:** Experimental design showing the rotational allocation of infested patch‐size treatments across three brood frames and six sequential experimental time points in ten selected honey bee colonies. Frames A–C were permanently assigned to the 8‐, 5‐, and 10‐cell infested patch treatments, respectively. Each infested cell received one live female *Varroa destructor* ; therefore, the treatments represent infested patch size (5, 8, or 10 neighboring cells). At each time point, the designated frame received its assigned infested patch treatment, with an equal number of sham‐manipulated cells on the opposite side of the same frame. The treatment sequence was rotated across six sequential time points (TP1–TP6), each separated by 72 h. Each colony received each of the three patch‐size treatments twice during the six time points. The treatment sequence was balanced across colonies to reduce potential time‐point and treatment‐order effects. Frames remained in their original colonies throughout the experiment. TP1–TP6 correspond to Days 0, 3, 6, 9, 12, and 15, respectively, given the 72 h postinoculation endpoint. A = 8‐cell patch; B = 5‐cell patch; C = 10‐cell patch.

## Data Availability

All relevant data are within the manuscript and its Supporting Information files.
