# Supplementary material for: Varroa‐Sensitive Hygiene (VSH) as a Potential Contributor to the Natural Survival of Ethiopian Honey Bees (Apis mellifera simensis) Against Varroa destructor Mite
Source: Ecol Evol. 2026 Oct 16;16(10):e74461. doi: 10.1002/ece3.74461 (PMC13673401; doi:10.1002/ece3.74461)
Supplement: Supplementary file 1 [file ECE3-16-e74461-s002.docx]

**Table S1.** Experimental design showing the rotational allocation of infested patch-size treatments across three brood frames and six sequential experimental time points in ten selected honey bee colonies. Frames A, B, and C were permanently assigned to the 8-, 5-, and 10-cell infested patch treatments, respectively. Each infested cell received one live female *Varroa destructor*; therefore, the treatments represent infested patch size (5, 8, or 10 neighbouring cells). At each time point, the designated frame received its assigned infested patch treatment, with an equal number of sham-manipulated cells on the opposite side of the same frame. The treatment sequence was rotated across six sequential time points (TP1–TP6), each separated by 72 h. Each colony received each of the three patch-size treatments twice during the six time points. The treatment sequence was balanced across colonies to reduce potential time-point and treatment-order effects. Frames remained in their original colonies throughout the experiment. TP1–TP6 correspond to Days 0, 3, 6, 9, 12, and 15, respectively, given the 72 hours post-inoculation endpoint. A = 8-cell patch; B = 5-cell patch; C = 10-cell patch. Top of FormBottom of Form

| **Colony** | **TP1 (Day 0)** | **TP2 (Day 3)** | **TP3 (Day 6)** | **TP4 (Day 9)** | **TP5 (Day 12)** | **TP6 (Day 15)** |
| --- | --- | --- | --- | --- | --- | --- |
| **1** | 10 (C) | 8 (A) | 5 (B) | 10 (C) | 8 (A) | 5 (B) |
| **2** | 8 (A) | 5 (B) | 10 (C) | 8 (A) | 5 (B) | 10 (C) |
| **3** | 5 (B) | 10 (C) | 8 (A) | 5 (B) | 10 (C) | 8 (A) |
| **4** | 10 (C) | 8 (A) | 5 (B) | 10 (C) | 8 (A) | 5 (B) |
| **5** | 8 (A) | 5 (B) | 10 (C) | 8 (A) | 5 (B) | 10 (C) |
| **6** | 5 (B) | 10 (C) | 8 (A) | 5 (B) | 10 (C) | 8 (A) |
| **7** | 10 (C) | 8 (A) | 5 (B) | 10 (C) | 8 (A) | 5 (B) |
| **8** | 8 (A) | 5 (B) | 10 (C) | 8 (A) | 5 (B) | 10 (C) |
| **9** | 5 (B) | 10 (C) | 8 (A) | 5 (B) | 10 (C) | 8 (A) |
| **10** | 10 (C) | 8 (A) | 5 (B) | 10 (C) | 8 (A) | 5 (B) |
