# Supplementary material for: Varroa‐Sensitive Hygiene (VSH) as a Potential Contributor to the Natural Survival of Ethiopian Honey Bees (Apis mellifera simensis) Against Varroa destructor Mite
Source: Ecol Evol. 2026 Oct 16;16(10):e74461. doi: 10.1002/ece3.74461 (PMC13673401; doi:10.1002/ece3.74461)
Supplement: Supplementary file 2 [file ECE3-16-e74461-s004.docx]

\# R v4.5.1

\# The purpose of this script is to analyse data and plot figures for the manuscript “*Varroa*-sensitive hygiene (VSH) as a potential contributor to the natural survival of Ethiopian honey bees (*Apis mellifera simensis*) against *Varroa destructor* mite” Tsegaye et al. submitted for publication.

######################################

**# General hygienic behaviour data #**

######################################

**# load packages**

library(carData)

library(car)

library (dplyr)

library(ggplot2)

library (agricol)

library(psych)

*#*	***Import the dataset in R*** *#*

my = read.csv("data.csv", header = TRUE, sep = ",")

*#*	***View the data***	*#*

View(data)

*#*	***Attach the data***	*#*

attach(data)

*#* ***Calculate summary statistics for each time point***	*#*

*data_summary <- data %>%*

*group_by(time) %>%*

*summarise(*

*mean = mean(percent),*

*se = sd(percent) / sqrt(n()),*

*lower = mean - 1.96 * se,*

*upper = mean + 1.96 * se,*

*n = n()*

*)*

*#*	***Compute variance among colonies for each time point: 24 h or 48 h***	*#*

var_24 <- var(ze$percent[ze$time == "24h"])

var_48 <- var(ze$percent[ze$time == "48h"])

cat("Variance at 24h:", var_24, "\n")	# Should be 53.30

cat("Variance at 48h:", var_48, "\n")	# Should be 2.70

*#* ***Draw spaghetti plot showing variability in the general hygienic behaviour among honey bee colonies at 24 hours and 48 hours*** *#*

*p_simple <- ggplot(data, aes(x = time, y = percent, group = Colony)) +*

*geom_line(aes(color = Colony)) +*

*geom_point(aes(color = Colony)) +*

*geom_hline(yintercept = 90, linetype = "dashed", color = "red") +*

*labs(title = "Hygienic behavior", x = "Time", y = "Cells removed (%)") +*

*theme_minimal()*

*print(p_simple)*

##############################################################################

**# VSH data.csv#**

##############################################################################

\# -----------------------------------------------------------------------------

\# **1. Load required packages**

\# -----------------------------------------------------------------------------

library(mclogit)

library(nnet)

library(sandwich)

library(lmtest)

library(carData)

library(car)

\# -----------------------------------------------------------------------------

\# **2. Import and prepare data**

**# -----------------------------------------------------------------------------**

\# **Import dataset**

my <- read.csv("data.csv", header = TRUE, sep = ",")

\# **Check structure**

str(my)

\# **View data**

View(my)

\# -----------------------------------------------------------------------------

\# **3. Prepare factors and experimental units**

\# -----------------------------------------------------------------------------

\# **Convert all categorical variables to factors**

my$Colony <- as.factor(my$Colony)

my$Timepoint <- factor(my$Timepoint, levels = c("TP1", "TP2", "TP3", "TP4", "TP5", "TP6"))

my$PatchSize <- factor(my$PatchSize, levels = c("5mites", "8mites", "10mites"))

my$ManipulationGroup <- factor(my$ManipulationGroup, levels = c("control", "infested"))

my$FrameID <- as.factor(my$FrameID)

\# **Create patch identifier for clustering**

my$PatchID <- interaction(my$Colony, my$FrameID)

\# **Create experimental unit identifier (Colony × Timepoint × FrameID)**

my$ExpUnit <- interaction(my$Colony, my$Timepoint, my$FrameID)

\# **Check number of unique experimental units**

length(unique(my$ExpUnit)) # Should be 60

\# -----------------------------------------------------------------------------

\# **4. Fit multinomial mixed-effects model (Primary model)**

**# -----------------------------------------------------------------------------**

\# **Model: Random intercept at experimental unit level**

model_expunit <- mblogit(

cbind(UC, CO, BMR, MR) ~ ManipulationGroup * PatchSize + Timepoint,

random = ~ 1 | ExpUnit,

data = my,

method = "MQL"

)

\# **Check convergence and summary**

summary(model_expunit)

\# --------------------------------------------------------------------------------------------------------------

\# **5. Fit reduced models for omnibus significance testing by comparing model deviances**

\# ---------------------------------------------------------------------------------------------------------------

\# **Model without ManipulationGroup**

model_no_group <- mblogit(

cbind(UC, CO, BMR, MR) ~ PatchSize + Timepoint,

random = ~ 1 | ExpUnit,

data = my,

method = "MQL"

)

\# **Model without PatchSize**

model_no_patch <- mblogit(

cbind(UC, CO, BMR, MR) ~ ManipulationGroup + Timepoint,

random = ~ 1 | ExpUnit,

data = my,

method = "MQL"

)

\# **Model without Timepoint**

model_no_time <- mblogit(

cbind(UC, CO, BMR, MR) ~ ManipulationGroup * PatchSize,

random = ~ 1 | ExpUnit,

data = my,

method = "MQL"

)

\# **Model without interaction (additive model)**

model_no_int <- mblogit(

cbind(UC, CO, BMR, MR) ~ ManipulationGroup + PatchSize + Timepoint,

random = ~ 1 | ExpUnit,

data = my,

method = "MQL"

)

\# **Null model (intercept only)**

null_model <- mblogit(

cbind(UC, CO, BMR, MR) ~ 1,

random = ~ 1 | ExpUnit,

data = my,

method = "MQL"

)

\# -----------------------------------------------------------------------------

\# **6. Likelihood Ratio Tests (Analysis-of-deviance)**

\# -----------------------------------------------------------------------------

\# **Test 1: ManipulationGroup**

lrt_group <- anova(model_expunit, model_no_group, test = "Chisq")

print(lrt_group)

\# **Test 2: PatchSize**

lrt_patch <- anova(model_expunit, model_no_patch, test = "Chisq")

print(lrt_patch)

\# **Test 3: Timepoint**

lrt_time <- anova(model_expunit, model_no_time, test = "Chisq")

print(lrt_time)

\# **Test 4: ManipulationGroup × PatchSize interaction**

lrt_int <- anova(model_expunit, model_no_int, test = "Chisq")

print(lrt_int)

\# **Test 5: Overall model (full vs null)**

lrt_overall <- anova(model_expunit, null_model, test = "Chisq")

print(lrt_overall)

\# -----------------------------------------------------------------------------

**# 7. Extract LRT results for summary table**

\# -----------------------------------------------------------------------------

**# Helper function to extract values**

extract_deviance <- function(anova_result) {

deviance <- abs(anova_result$Deviance[2])

df <- abs(anova_result$Df[2])

p_value <- anova_result$`Pr(>Chi)`[2]

return(data.frame(

Deviance = deviance,

Df = df,

P_value = p_value

))

}

**# Extract for each test**

group_vals	<- extract_deviance(lrt_group)

patch_vals	<- extract_deviance(lrt_patch)

time_vals	<- extract_deviance(lrt_time)

int_vals	<- extract_deviance(lrt_int)

overall_vals <- extract_deviance(lrt_overall)

**# Create summary table**

results_table <- data.frame(

Effect = c(

"ManipulationGroup",

"PatchSize",

"Timepoint",

"ManipulationGroup × PatchSize",

"Overall model"

),

Deviance_difference = c(

group_vals$Deviance,

patch_vals$Deviance,

time_vals$Deviance,

int_vals$Deviance,

overall_vals$Deviance

),

df = c(

group_vals$Df,

patch_vals$Df,

time_vals$Df,

int_vals$Df,

overall_vals$Df

),

P_value = c(

group_vals$P_value,

patch_vals$P_value,

time_vals$P_value,

int_vals$P_value,

overall_vals$P_value

)

)

**# Format p-values**

results_table$P_value_formatted <-

format(results_table$P_value,

scientific = TRUE,

digits = 3)

**# Print results table**

print(results_table)

\# --------------------------------------------------------------------------------------------

\# **8. Pairwise comparisons: Control vs Infested at each patch size**

**Manual extraction using coefficients and variance-covariance matrix**

\# --------------------------------------------------------------------------------------------

**# Get coefficients and variance-covariance matrix**

b <- coef(model_expunit)

V <- vcov(model_expunit)

**# Function to calculate control vs infested contrasts for each outcome and patch size**

get_contrast <- function(outcome, patch) {

**# Main control-vs-infested coefficient**

main_name <- paste0(outcome, "~ManipulationGroupinfested")

**# Interaction term for patch size 8 or 10**

interaction_name <- if (patch == "5") {

NULL

} else {

paste0(outcome, "~ManipulationGroupinfested:PatchSize", patch, "mites")

}

**# Create contrast vector**

L <- rep(0, length(b))

names(L) <- names(b)

L[main_name] <- 1

if (!is.null(interaction_name)) {

L[interaction_name] <- 1

}

**# Estimate**

estimate <- sum(L * b)

**# Standard error using the covariance matrix**

SE <- sqrt(as.numeric(t(L) %*% V %*% L))

**# Wald z test**

z <- estimate / SE

**# Raw p-value**

p <- 2 * pnorm(abs(z), lower.tail = FALSE)

**# Odds ratio and 95% CI**

OR <- exp(estimate)

CI_low <- exp(estimate - qnorm(0.975) * SE)

CI_high <- exp(estimate + qnorm(0.975) * SE)

data.frame(

Outcome = outcome,

PatchSize = patch,

Log_OR = estimate,

SE = SE,

OR = OR,

CI_low = CI_low,

CI_high = CI_high,

z = z,

p = p

)

}

**# Calculate all 9 contrasts (3 behaviours × 3 patch sizes)**

results <- rbind(

get_contrast("CO", "5"),

get_contrast("BMR", "5"),

get_contrast("MR", "5"),

get_contrast("CO", "8"),

get_contrast("BMR", "8"),

get_contrast("MR", "8"),

get_contrast("CO", "10"),

get_contrast("BMR", "10"),

get_contrast("MR", "10")

)

**# Bonferroni correction across all 9 planned comparisons**

results$p_Bonferroni <- p.adjust(results$p, method = "bonferroni")

**# Print results**

print(results)

\# -----------------------------------------------------------------------------

\# **9. Model-predicted probabilities and composite responses across TP1-TP6**

\# -----------------------------------------------------------------------------

\# **Create new data for all combinations of ManipulationGroup**

\# **PatchSize, and Timepoint**

newdat <- expand.grid(

ManipulationGroup = levels(my$ManipulationGroup),

PatchSize = levels(my$PatchSize),

Timepoint = levels(my$Timepoint)

)

\# **Set ExpUnit to a valid level for prediction**

newdat$ExpUnit <- my$ExpUnit[1]

\# **Predict model-based probabilities**

pred <- predict(

model_expunit,

newdata = newdat,

type = "response"

)

\# **Create prediction table**

pred_df <- cbind(

newdat[, c("ManipulationGroup", "PatchSize", "Timepoint")],

as.data.frame(pred)

)

**# Add composite responses**

pred_df$Removal <- pred_df$BMR + pred_df$MR

pred_df$Uncapping <- pred_df$CO +

pred_df$BMR +

pred_df$MR

**# Average predicted probabilities across the six timepoints**

**# for each ManipulationGroup × PatchSize combination**

pred_summary <- aggregate(

cbind(UC, CO, BMR, MR, Removal, Uncapping) ~

ManipulationGroup + PatchSize,

data = pred_df,

FUN = mean

)

**# Convert probabilities to percentages**

pred_summary[, c("UC", "CO", "BMR", "MR",

"Removal", "Uncapping")] <-

pred_summary[, c("UC", "CO", "BMR", "MR",

"Removal", "Uncapping")] * 100

**# Print model-predicted probabilities reported in the manuscript**

print(pred_summary)
