# Supplementary material for: Varroa‐Sensitive Hygiene (VSH) as a Potential Contributor to the Natural Survival of Ethiopian Honey Bees (Apis mellifera simensis) Against Varroa destructor Mite
Source: Ecol Evol. 2026 Oct 16;16(10):e74461. doi: 10.1002/ece3.74461 (PMC13673401; doi:10.1002/ece3.74461)
Supplement: Supplementary file 3 [file ECE3-16-e74461-s005.docx]

This README file was generated on 2026-September-07 by Beatrice T. Nganso

**GENERAL INFORMATION**

**Goal of the study**:

To characterize general hygienic behaviour (GHB) and *Varroa*-sensitive hygiene (VSH) responses in Apis mellifera simensis colonies from Ethiopia, including the effects of infested brood patch size on VSH behavioural responses. The study specifically assessed GHB using the pin-killed brood assay and VSH responses to artificially mite-infested and sham-manipulated worker brood cells.

**Title of Dataset:**

General hygienic behaviour and *Varroa*-sensitive hygiene responses of *Apis mellifera simensis* colonies in Ethiopia.

**Authors Information:**

A. Principal Investigator Contact Information

Name: Beatrice T. Nganso

Institution: International Centre of Insect Physiology and Ecology (*icipe*)

Address: Nairobi, Kenya

Email: [bnganso@icipe.org/ngansobeatrice@gmail.com](mailto:bnganso@icipe.org/ngansobeatrice@gmail.com)

B. Co-Investigator Contact Information

Name: Alemu Tsegaye

Institution: International Centre of Insect Physiology and Ecology (*icipe*)

Address: Nairobi, Kenya

Email: [alemutsg@gmail.com](mailto:alemutsg@gmail.com)

**Date of data collection (single Date, range, approximate Date):**

August-November 2024

**Geographical location of data collection:**

Jinkaba Experimental Apiary, Sekota Dryland Agricultural Research Center (SDARC), Amhara Region, Ethiopia. Coordinates: 12°41′01.02″ N, 39°00′31.53″ E

**Information about funding sources that supported the collection of the data**:

Data collection in Ethiopia and analysis at *icipe* was funded by the Mastercard Foundation through the More Young Entrepreneurs in Silk and Honey (MOYESH) Programme. Alemu T. Adane acknowledges the Bahir Dar University and Amahara Agricultural Research Institute (ARARI), FSRP Project for funding his PhD research work and studies.

**SHARING/ACCESS INFORMATION**

1. **Licenses/restrictions placed on the data**: None
2. **Links to publications that cite or use the data**:

Tsegaye, A., Aynalem, T., Taye, M., Ayalew, W., Jenberie, A., Chege, M., Tefera, T., & Nganso, B. (2024). *Varroa*-sensitive hygiene (VSH) as a potential contributor to the natural survival of Ethiopian honey bees (*Apis mellifera simensis*) against *Varroa destructor* mite

3. **Links to other publicly accessible locations of the data:** None
4. **Links/relationship to ancillary data sets:** None
5. **Was data derived from another source**? No

If yes, list source(s): NA

6. **Recommended citation for this dataset:**

Tsegaye, A., Aynalem, T., Taye, M., Ayalew, W., Jenberie, A., Chege, M., Tefera, T., & Nganso, B. (2024). *Varroa*-sensitive hygiene (VSH) as a potential contributor to the natural survival of Ethiopian honey bees (*Apis mellifera simensis*) against *Varroa destructor* mite

**DATA AND FILE OVERVIEW**

1. **File List:**
A) Data used for quantifying general hygienic behaviour at 24 h and 48 h
B) Data used for comparing the VSH responses between control and treatment at varying patch size treatment (five, eight, or ten neighbouring infested cells), and time of manipulation
2. **Relationship between files, if important**: None
3. **Additional related data collected that was not included in the current data package:** None
4. **Are there multiple versions of the dataset?** No

A. If yes, name of file(s) that was upDated: NA

i. Why was the file upDated? NA

ii. When was the file upDated? NA

#########################################################################

**DATA-SPECIFIC INFORMATION FOR: General hygienic behaviour data.csv**

These data were used to quantify general hygienic behaviour at 24 h and 48 h in colonies of *A. m. simensis* to select colonies exhibiting more than 90% removal rate at 24 hours. Box plots with superimposed jitter were used to illustrate the distributions and highlight variation at the colony level using the dplyr (Wickham et al., 2026) and ggplot2 (Wickham, 2016) packages.

1. Number of variables: 5

2. Number of cases/rows: 41

3. Variable List:

* Name of variables: Colony, total, time, removed, percent

* Year: Year of the study (2024)

* Patch: Brood fully removed following pin-killed assay where the variables collected

* Date: Date of sample collection (September 2022)

4. Missing data codes: None

5. Specialized formats or other abbreviations used: None

#########################################################################

**DATA-SPECIFIC INFORMATION FOR: VSH data.csv**

These data were used to compare the four VSH behavioural responses between the control and treatment under varying patch size treatment and time of manipulation as fixed effects, alongside with the interaction term between treatment group and patch size treatment. A random intercept was included for the frame-pair unit, defined as the combination of colony, time of manipulation, and frame ID (colony × time of manipulation × frame ID), to account for the pairing of control and infested patches on the same frame and repeated observations within colonies..

1. Number of variables: 10

2. Number of cases/rows: 121

3. Variable List:

* Name of variables: Colony, timepoint, PatchSize, FrameID, ManipulationGroup, totalcells, BMR, MR, CO and UC

* Year: Year of the study (2024)

*Patch: The four VSH behavioural responses (BMR, MR, CO and UC) were collected in control and treatment groups

4. Missing data codes: None

5. Specialized formats or other abbreviations used: None
